# A Cyber–Physical Production System for the Integrated Operation and Monitoring of a Continuous Manufacturing Train for the Production of Monoclonal Antibodies

**DOI:** 10.3390/bioengineering11060610

**Published:** 2024-06-13

**Authors:** Garima Thakur, Saxena Nikita, Vinesh Balakrishnan Yezhuvath, Venkata Sudheendra Buddhiraju, Anurag S. Rathore

**Affiliations:** 1 Department of Chemical Engineering, Indian Institute of Technology Delhi, Hauz Khas, Delhi 110016, India; 2 TCS Research, Tata Consultancy Services Limited, Pune 411057, Indiavenkata.buddhiraju@tcs.com (V.S.B.)

**Keywords:** cyber–physical production system, monoclonal antibody, continuous bioprocessing, automation, process monitoring, process control

## Abstract

The continuous manufacturing of biologics offers significant advantages in terms of reducing manufacturing costs and increasing capacity, but it is not yet widely implemented by the industry due to major challenges in the automation, scheduling, process monitoring, continued process verification, and real-time control of multiple interconnected processing steps, which must be tightly controlled to produce a safe and efficacious product. The process produces a large amount of data from different sensors, analytical instruments, and offline analyses, requiring organization, storage, and analyses for process monitoring and control without compromising accuracy. We present a case study of a cyber–physical production system (CPPS) for the continuous manufacturing of mAbs that provides an automation infrastructure for data collection and storage in a data historian, along with data management tools that enable real-time analysis of the ongoing process using multivariate algorithms. The CPPS also facilitates process control and provides support in handling deviations at the process level by allowing the continuous train to re-adjust itself via a series of interconnected surge tanks and by recommending corrective actions to the operator. Successful steady-state operation is demonstrated for 55 h with end-to-end process automation and data collection via a range of in-line and at-line sensors. Following this, a series of deviations in the downstream unit operations, including affinity capture chromatography, cation exchange chromatography, and ultrafiltration, are monitored and tracked using multivariate approaches and in-process controls. The system is in line with Industry 4.0 and smart manufacturing concepts and is the first end-to-end CPPS for the continuous manufacturing of mAbs.

## 1. Introduction

The continuous manufacturing of biotherapeutics, a focal point for both academic and industrial entities in the biotherapeutic sector, has witnessed remarkable advancements over the past decade, particularly in technologies aimed at enabling the continuous production of various mammalian and microbial biotherapeutics [1]. Notable progress includes the development and implementation of perfusion cell culture systems for high-titre biomolecule production upstream, alongside the emergence of various clarification systems like continuous centrifugation, alternating tangential flow filtration, and acoustic wave separation, facilitating integration between upstream and downstream processes [2]. The introduction of versatile chromatography setups and customized flow reactors has further expanded our capability for continuous processing, including achieving low pH conditions and executing reactions previously confined to batch mode. Innovative membrane modules for continuous formulation have also emerged, boasting high concentration factors and efficient buffer exchange. However, the transition from batch to continuous mode presents unique challenges, notably in the handling of real-time deviation without interrupting the process flow, necessitating robust sets of online or at-line analytical tools coupled with automated control strategies [3]. It is critical to achieve process digitalization and leverage computational approaches such as statistical process control, machine learning, and artificial intelligence to successfully operate continuous manufacturing processes.

Cyber–physical production systems (CPPSs) are a key concept in developing smart manufacturing processes [4]. The development of CPPSs have influenced multiple fields related to technology and engineering including transportation, defence, energy, food, petrochemicals, and healthcare [5]. The biopharmaceutical industry is currently lagging behind other industries in implementing Manufacturing 4.0; however, it has adopted continuous manufacturing concepts [6]. However, there are still challenges in process automation, integration, and control that have thus far been a roadblock preventing the widespread adoption of continuous processing in the biopharmaceutical industry. CPPSs provide an effective tool for overcoming these automation and control challenges and developing reliable and robust frameworks for continuous processing [7,8]. In this work, we showcase the development of a CPPS system for the continuous manufacturing of monoclonal antibodies (mAbs), which are currently the largest class of biotherapeutics and have a well-established manufacturing process platform that is suitable for adapting into a continuous processing platform. There are three major elements of CPPSs, i.e., “connectedness”, “intelligence”, and “responsiveness”, and all these elements work together to execute, monitor, and control an end-to-end continuous process [4,5]. Integrating the system with digital twins can be an added advantage. The adoption of digital solutions has further enabled the implementation of Industry 4.0 concepts [6].

CPPSs can also be a powerful enabler of continued process verification (CPV), which is an approach followed by biopharmaceutical companies to continuously monitor and validate manufacturing plants and processes, with the aim of ensuring consistent product quality [9]. CPV leverages knowledge of the relationships between critical process parameters (CPPs) and critical quality attributes (CQAs) [10]. CPV continues to evolve as more data are generated and can be leveraged as the framework for data collection, cleaning, storage, and retrieval, with functionality to take into account the diverse types and frequencies of data acquired during the operation process from various in-line, at-line, or off-line sensors. Though well-established for batch-mode manufacturing, CPV approaches specific to the continuous manufacturing of biopharmaceuticals have thus far gained minimal attention [11,12]. In 2014, a consortium of biopharmaceutical companies published a case study on CPV for mAb manufacturing processes [13]. However, the recommendations in the plan were for batch-style manufacturing with individual unconnected unit operations. In 2019 and 2021, the US FDA published two draft guidance documents emphasizing continuous manufacturing [14,15]. There is a need for case studies showcasing the use of CPPSs for continuous processes that can enable process monitoring at the level required for CPV. Various groups have worked on different aspects of the integration [16], automation [17,18], monitoring [19] and control [20,21,22] of biopharmaceutical processes. However, this is the first work which has a focus on developing an end-to-end CPPS development case study, including hardware–software integration, data acquisition and storage, a data historian, multivariate data analysis, and surge-tank-based process control within a single integrated framework.

In this paper, we use a lab-scale continuous train for mAb production to develop a CPPS framework. Since continuous processes require scale-up in terms of campaign length rather than operation volume, with columns typically of a scale of <1 L, they generate only moderate scale-up considerations compared to batch processes with single-column large-scale purifications. Thus, a lab-scale process can provide useful insights into many of the key elements of a CPPS. A programmable logic controller (PLC) and supervisory control and data acquisition software (SCADA) are employed for automation and digitalization to implement the unified process supervisory framework, which consists of three key modules—connectedness, intelligence, and responsiveness—that deal with the automation and integration of equipment with a distributed control system to facilitate data acquisition and the execution of control actions, handle real-time data analysis during the operation process to generate useful insights, and manage control actions and process adaptations to provide real-time alarms and corrective actions when integrated further with digital twins. Overall, the CPPS framework enables effective integration, automation, and digitalization of continuous biopharmaceutical manufacturing, as discussed and validated in Section 2, Section 3 and Section 4.

## 2. Materials and Methods

### 2.1. Materials

In this study, an immunoglobulin G1 (IgG1) antibody was used, with a molecular weight of approximately 150 kDa (kilodaltons) and a pI (isoelectric point) of 8.1. The research utilized 20 L of harvested cell culture fluid from a major Indian biopharmaceutical manufacturer, containing the monoclonal antibody (mAb) at a concentration of 5.5 g/L. Analytical-grade chemicals, including glycine, Tris base, acetic acid (CH_3_COOH), sodium chloride (NaCl), sodium dihydrogenphosphate (NaH_2_PO_4_), disodium hydrogen phosphate (Na_2_HPO_4_), sodium hydroxide (NaOH), hydrochloric acid (HCl), and histidine, were procured from Merck (Mumbai, India). Buffer solutions were prepared using deionized water, and all solutions underwent filtration through a 0.2 μm filter.

### 2.2. Methods for Unit Operations

**Figure 1:**
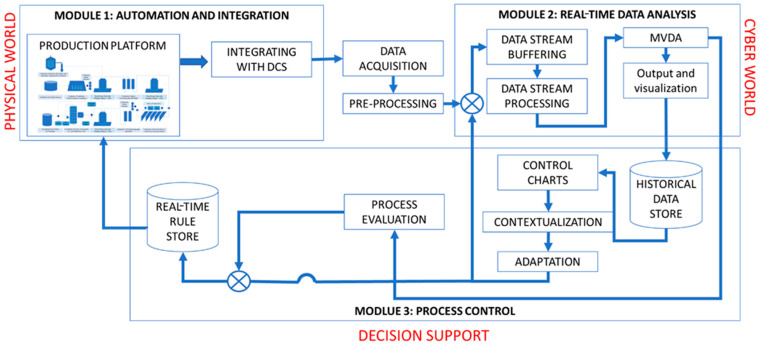
Overview of cyber–physical production system framework.

**Figure 2:**
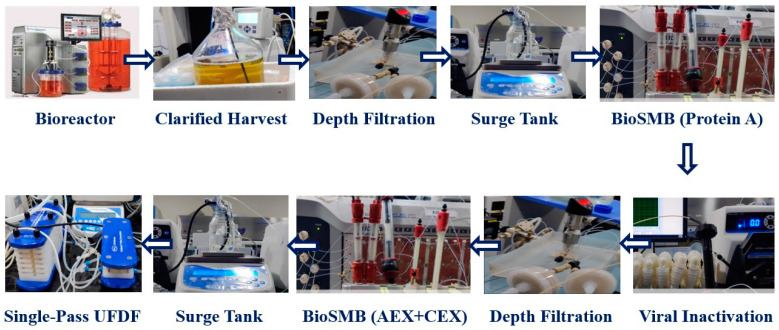
Schematic showing laboratory setup of the continuous mAb production platform.

A lab-scale continuous monoclonal antibody (mAb) production train was established. The continuous process involved fed-batch cell culture harvest, clarification using acoustic wave separation, and depth filtration, followed by Protein A chromatography, viral inactivation, sterile filtration, cation exchange chromatography, single-pass ultrafiltration, and single-pass diafiltration. The overarching CPPS is illustrated in Figure 1 and the continuous train setup is illustrated in Figure 2. The continuous clarification utilized a Pall (New York, NY, USA) Cadence^TM^ Acoustic Wave Separator (AWS) with four acoustic chambers in series for a 90% reduction in total cell density [23]. Continuous dead-end filtration employed an in-house-developed filtration skid, incorporating a customized solenoid valve to connect three filters in parallel [24]. The skids were strategically placed between clarification and Protein A chromatography for depth filtration and between viral inactivation and polishing chromatography for sterile filtration. Continuous Protein A and cation exchange (CEX) chromatography were performed on a Pall Cadence^TM^ BioSMB system using MabSelectSureTM resin (GE Healthcare Life Sciences, Stockholm, Sweden) and Poros HS resin (Applied Biosystems, Waltham, MA, USA), respectively. Both chromatography steps were conducted on the same BioSMB system, integrated with a programmable gradient pump and controlled through a Python layer. The continuous Protein A chromatography was executed on a Pall Cadence^TM^ BioSMB system with three columns (16.7 mL volume each) packed with MabSelectSureTM resin. A Periodic Counter Current (PCC) method was employed. Continuous cation exchange chromatography (CEX) was also performed on the same BioSMB system using Poros HS resin and a pH gradient. Continuous viral inactivation utilized a low pH hold in a coiled flow inversion reactor (CFIR) with in-line pH adjustment [25]. Continuous ultrafiltration and diafiltration (UF-DF) employed the Pall (New York, USA) Cadence^TM^ In-Line Concentrator (ILC) and In-Line Diafiltration (ILD) modules, respectively [26].

### 2.3. Developing a Cyber–Physical Production System (CPPS)

The first step in developing a CPPS framework is to establish digital connections between multiple hardware and software units that originally operated autonomously and independently. These connections can be in a horizontal direction, such as between different unit equipment in the manufacturing facility, or vertically, such as between multiple levels of operational intelligence, such as logistics for raw material sourcing, the transport of drug substance intermediates between different facilities, or the distribution of the final product to the end user [27]. In this work, we focus on only a horizontal CPPS between different unit operations in a single integrated continuous bioprocessing facility. Data are acquired by interlinking multiple production equipment, and access is allowed to both live data, past data from the current manufacturing campaign, and past data from former manufacturing campaigns. This facilitates the evaluation of the process based on both real-time and historical data and can allow for the prediction of future trends in the process, which can be implemented for controlling process deviations. Figure 1 illustrates the overall architecture of the CPPS framework, integrating all three modules with a free flow of information between modules. The figure illustrates a generic and flexible platform for ease of implementation and adoption. The general structure of each of the three modules is explained below.

#### 2.3.1. Module 1 (“Connectedness”): Automation and Integration

The “Connectedness” module includes the process equipment and sensors, all of which are automated using a PLC, which was, in this case, a distributed control system (DCS) unit from Siemens (Munich, Germany). The PLC helps in simplifying complex production platforms and reduces setup time, while proving better performance and economy. All the equipment is connected to the PLC over a local intranet to facilitate the scheduling of the different steps in the manufacturing process using linked triggers. Overall, this module maintains system topology, wherein a generic communication protocol is employed to make the data uniform and easily interpretable by the second and third module for data analytics and control decisions, respectively. 

#### 2.3.2. Module 2 (“Intelligence”): Real-Time Data Analytics

The “Intelligence” module is the real-time data analytics framework, including a data historian, pre-processing algorithms, and a multivariate data analytics module for tracking the current process’s trajectory. The first component of this module is the data stream buffering component, which has the ability to handle large volumes of data from all the equipment and sensors simultaneously, with high frequency, passing the data onwards to the next component without undue delays. The next component is data processing, where the data are analysed and trended using various algorithms including statistical upper and lower limit detection [28,29]. The multivariate data components serve to capture the key process information and reduce data noise, helping to identify patterns, faults, and deviations that can lead to quality issues or equipment breakdown [30]. This module also contains a data visualization component that plots graphs showing the trends of the process variables and MVDA components to maximize their interpretability by the human operator. The data analytics outputs are also fed to the third module to update the control rules and logic and inform control actions based on the current state of the process.

#### 2.3.3. Module 3 (“Responsiveness”): Process Control 

The “Responsiveness” module is the knowledge management component, which brings all the process data into one context for control and contains control strategies that can be executed in response to the data analytics, including digital twins and surge-tank-based control strategies. While data analytics deals with the actual data analysis of the process, contextualization includes domain knowledge in addition to purely data-driven insights. This module takes into account raw data, historical data, and the knowledge generated by the second module. The data are passed into control algorithms including rule-based controls, feedback or feedforward control algorithms, digital twins, and surge-tank-based control strategies depending on the control developed for the end-to-end platform, and for individual unit operations. The module then passes instructions back to the first module to execute operations. It also adaptively updates the real-time rule store if required. Overall, this enables process quality to be maintained and prevents the propagation of deviations across unit operations, maintaining steady-state operation throughout the continuous platform. 

## 3. Results

**Figure 3:**
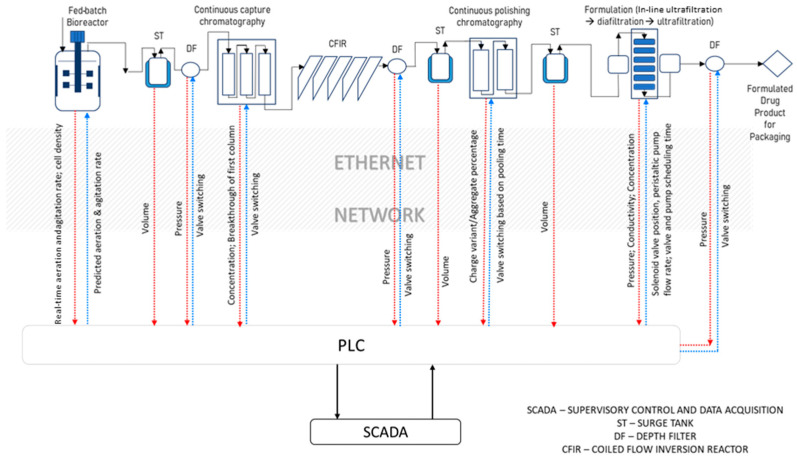
Integration of equipment with PLC and SCADA.

A continuous mAb purification platform comprising capture chromatography, viral inactivation, polishing chromatography, dead-end filtration, ultrafiltration, and diafiltration (Figure 2) was developed at lab scale, as reported in previous studies published by our group [22,23,24,26]. The types of input–output communication ports in the hardware (analogue or digital) and in the software (Modbus 485, RS-232, Modbus TCP/IP, Profibus DP or Ethernet TCP/IP) were listed as part of the process equipment and sensors, as given in Table 1. As stated above, the PLC was a DCS unit (Siemens). Based on this information, the next step is to design a PLC containing at least the minimum number of required ports such that it can be interfaced with all the equipment using a combination of unmanaged and Layer 3 switches via the ethernet, including the configuration of IP addresses and comm ports for proper communication. This integration with the PLC is the “industrial automation layer”, which includes PLC programming and process scheduling. This is linked to a data archive where all the real-time data are stored in the form of time-stamped key-value tables, as well as process scheduling instructions, as shown in Figure 3. Overall, the PLC is a key part of Module 1 and acts as a master controller, acquiring data and executing the control instructions acquired from all three modules of the CPPS, including scheduling instructions from the supervisory control and data acquisition (SCADA) orchestration layer in Module 1, MVDA-based alarms and equipment pause instructions from Module 2, and control strategies from Module 3. 

## 4. Results and Discussion

### 4.1. Process Data Collection and Continued Process Verification (CPV)

One of the key advantages offered by CPPS is that of CPV. CPV involves monitoring the commercial manufacturing of products to ensure product quality and thereby enables continuous improvement in production processes. As part of the Quality by Design paradigm, it is recommended that strong cause-and-effect relationships be established between CQAs and different process operating parameters, including classifying process parameters (PPs) such as critical parameters (CPPs), well-controlled critical parameters (WC-CPPs), key parameters (KPPs), and general parameters (GPPs) [3]. Once these are established, these parameters need to be monitored in order to flag deviations that could impact the CQAs throughout the manufacturing life cycle of the product.

**Figure 4:**
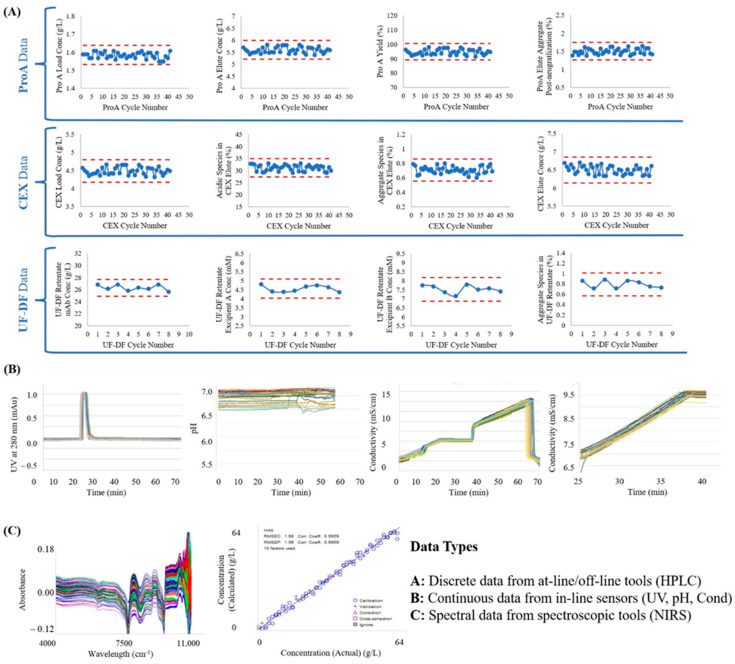
Different types of data acquired during the process run from process equipment sensors, external sensors, and analytical tools. Note: only data from the first 48 h are shown for clarity. In (**A**), red dashed lines show ±1 SD from the mean of the data in each chart. In (**B**,**C**), different coloured lines show the overlay of data from each cycle in the continuous processing train.

A major challenge faced while implementing CPV in integrated continuous manufacturing is establishing frameworks to acquire, store, and analyse the large amounts of data required for delivering CPV across all unit operations simultaneously and at a high frequency. Furthermore, variables formerly considered non-critical may become more important in continuous processing and need regular monitoring. In addition to CPPS providing automated data acquisition and analysis at high frequencies over long continuous campaigns, it maintains data integrity by assimilating multiple data sources into a single centralised historian that can ease data buffering, pre-processing, visualization, and analysis. For the mAb manufacturing process, the data (discrete, continuous, and spectral) from the process equipment’s sensors, external sensors, and analytical tools were acquired, stored in a time-stamped array, and processed differently, in line with the nature and purpose of the data (refer Supplementary S1 for pre-processing of spectral data). Figure 4A shows the discrete data measured by HPLC tracking CQAs for the major unit operations of the first 48 h of the steady-state operation of the continuous process. 

The continuous data were from in-line sensors, either belonging to the unit operation equipment or externally from the surge tanks between process steps. These types of data were stored with both time stamps as well as ID numbers corresponding to the cycle number of the respective chromatography or filtration step. Each cycle of chromatography is defined from the start of loading to the start of the next load, including the load, wash, elution, cleaning, and equilibration steps. Each cycle of filtration is defined as from the start of the feed pump to the pause of the feed pump, with or without cleaning cycles in between. Figure 4B shows an example of the continuous data acquired from the BioSMB for the CEX chromatography step, whereas Figure 4C shows the spectral data acquired during this process. These profiles were then used as the input for the MVDA models discussed in the next section.

### 4.2. Multivariate Data Analytics (MVDA)

**Figure 5:**
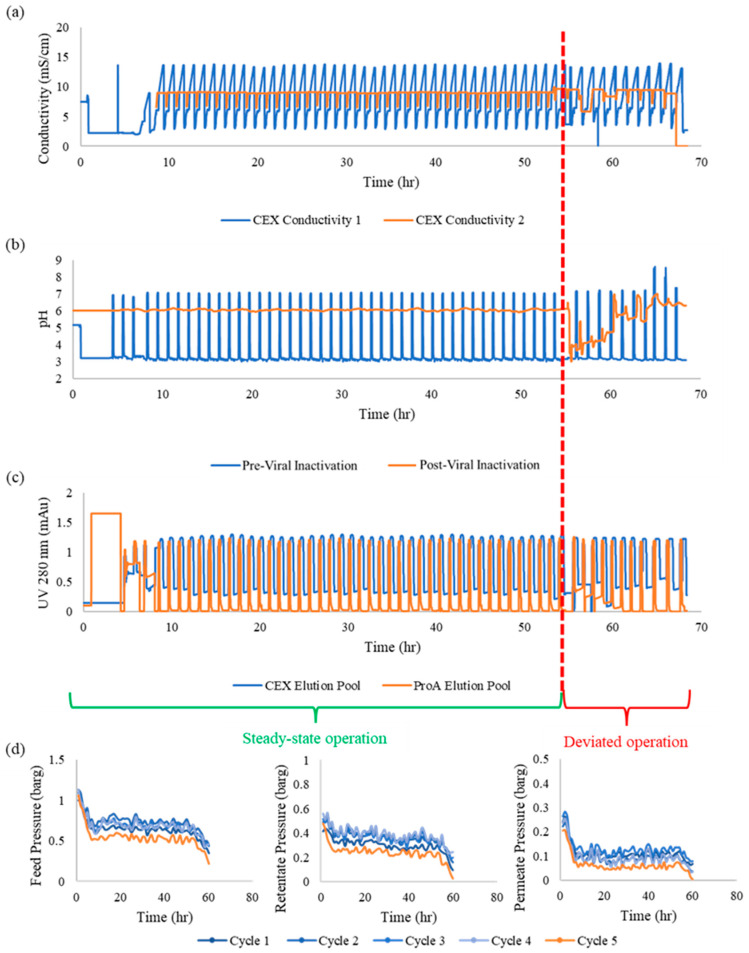
Continuous process data from (**a**–**c**) chromatography sensors and (**d**) filtration sensors. (**a**) shows conductivity profiles from sensors in the non-pool (blue) and pool (orange) outlet lines of the cation exchange chromatography column, showing the linear gradient conductivity increases across all the continuous process cycles. (**b**) shows the pH profiles in the protein A elution outlet (blue) and viral inactivation line (orange) showing the low-pH elution followed by neutralization. (**c**) shows the UV profiles of the CEX (blue) and Protein A (orange) elution pools, showing consistent elution peak collection across both chromatography operations. (**d**) shows the feed (left), retentate (middle) and permeate (right) pressure profiles across five cycles of UF-DF operation.

**Figure 6:**
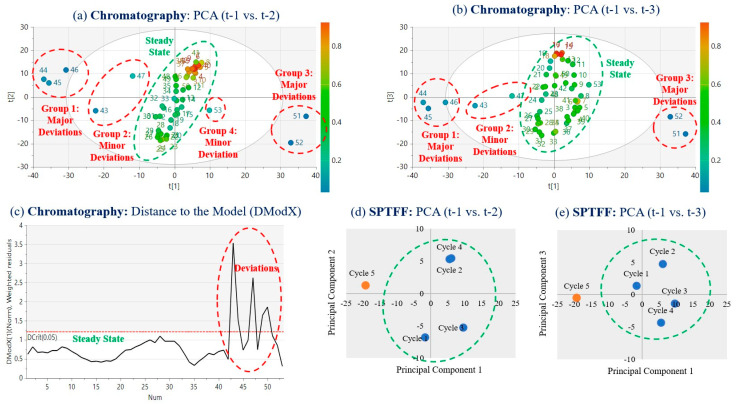
PCA analysis of chromatography and filtration data.

MVDA is a widely adopted tool in the biopharmaceutical industry. It is important for the CPPS to incorporate the functionality to carry out multivariate analyses, visually represent the data in MVDA plots, and refresh the analyses in real time. This enables the most recent data to be compared to the steady-state limits to identify deviations as and when they occur. Figure 5 shows the real-time data from all the in-line process sensors of the chromatography and filtration steps for 60 h of continuous operation. The MVDA algorithms were developed to group data points by cycle based on the time stamps at the beginning and end of each cycle. After this, principal component analysis (PCA) was employed to reduce data dimensionality (refer Supplementary S2 for pseudocode). The PCA plots for the data from the two primary downstream unit operations are given in Figure 5 and Figure 6. It is evident that the steady-state cycles are clustered within the centre of the latent variable space, while the deviated runs are outside the central cluster. Figure 6a shows the PCA plot for the first vs. second principal component, while Figure 6b shows the plot for the first vs. third principal component. Figure 6c shows the DModX score for each cycle, which captures the distance between the cycle data and the PCA model space. It can be seen in Figure 5a,b that the pH and pressure deviations started around cycle 43, which is away from the main cluster but still within the PCA space, and that the highly deviated cycles 44–46 are clear outliers, before cycle 47 again becomes close to a steady state. The process is stabilised in cycles 48–50, before deviations arise in cycles 51 and 52 which are partially recovered by cycle 53, which deviated only in Figure 6a and not in Figure 6b. Likewise, the deviated cycles are clearly visible on the DModX plot, where it can be seen that the process has moved out of its controlled state during the last 12 h of operation. Similarly, Figure 6d,e show the PCA plots of the first vs. second and first vs. third principal components for the filtration cycles, in which it can be seen that cycle 5 deviated as it is outside the PCA clusters in both plots.

### 4.3. Process Control in Response to Measured Data—Module 3

**Figure 7:**
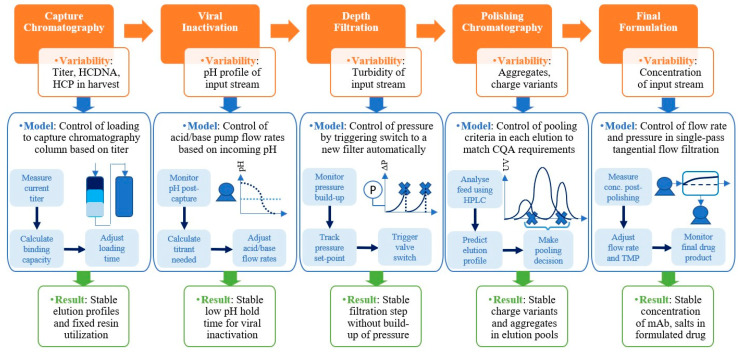
Summary of surge tank control strategies for handling process deviations.

Figure 7 shows the chromatography data obtained from the BioSMB during 48 h of steady-state operation, followed by 12 h with various deviations in the process that arose during operation. The process achieved a steady state with consistent UV, pH, and conductivity profiles from t = 7 h to t = 55 h, after which several process deviations arose and can be observed in the data. These deviations were of various types. First, there was an error in the pump that was used to supply Tris base in-line at the exit of the viral inactivation reactor, leading to pH deviations in the CEX load material and affecting the CEX elution profiles. The pH deviation started in cycle 43 and persisted until cycle 47 before the pump error was fixed and the pH data normalized. Another deviation occurring during these cycles was a pressure build-up in the ProA column, causing deviations in the UV data until the column was switched out for a standby column around the time of cycle 45. Yet another deviation occurred in cycle 44 in the CEX elution, in which there was an error in the conductivity gradient delivered by the BioSMB pump due to the emptying of the buffer tank. Finally, in cycles 51–52, there was an error in which some of the ProA cleaning buffer mistakenly entered the ProA elution stream due to the improper closing of BioSMB valves, leading to the pH spikes observed in these two cycles before the error was rectified. In order to effectively handle deviations without needing the entire continuous train to stop, automated real-time corrective measures are required.

These provide process safety in cases of errors in the process equipment, such as column failure, membrane rupture, pressure build-up, tubing leakage, and power loss, all of which require the relevant equipment unit to be paused for checks, repairs, cleaning, or replacement. In the event of these unpredictable errors, the surge tanks essentially act as reservoirs into which the incoming process material from the previous unit operation can continue to flow, without interruption mid-cycle, while the repairs are performed. Similarly, the subsequent unit operation can also continue until one cycle is complete, thus preventing the CQAs from fluctuating due to the error. This provides a significant amount of repair time in which equipment errors can be rectified without having to stop the entire continuous train. In order to implement surge-tank-based control strategies, a series of weighing balances need to be deployed to consistently track the volume of each surge tank, and different trigger flags need to be coded into the CPPS that can be deployed manually or automatically whenever an equipment error or failure is detected in the continuous train. As discussed, the continuous train was operated at a steady state for the first 48 h after start-up (7–55 h), after which some deviations arose in its final 12 h of operation. These deviations included pump errors, valve errors, tubing errors, and sensor errors. As shown in the MVDA plots in Figure 6, these deviations led to the continuous train shifting out of its steady-state range. In all of these cases, surge tank controls are an effective way to prevent the error from persisting across several cycles and risking the derailment of the stable continuous process, as we have shown in previous work [22,23,24,26]. Figure 7 illustrates some of the key surge-tank-based decisions that can be taken when such errors are detected. Table 2 lists the errors detected and the strategies deployed in the last 12 h of the run which helped to bring the process back on track. The surge tank control strategies are suitable for handling equipment errors and stability issues, in which the impacted volumes of material can be discarded and the equipment part repaired or replaced during the brief period of pause facilitated by the surge tanks. A detailed description of the corrective actions implemented by Module 3 in response to deviations is discussed further. This logic was coded into Module 3 of the CPPS using Python scripts developed in previous work [22,23,24,26].

### 4.4. ProA Chromatography Deviations

The first deviation in ProA chromatography (Deviation 1 in Table 2) was due to column pressure build-up causing leakage from the column inlet tubing. This triggered a pop-up window with an alarm message to alert the operator. At the same time, the loading into Protein A was switched into a standby safety Protein A column. The outlet valves for the deviated column were directed to waste. The previous unit operation continued on as normal as the weight of the surge tank was maintained due to an immediate switch of the load to a standby safety column.

The next deviation in ProA chromatography (Deviation 4 in Table 2) arose due to an error in the BioSMB control system, which hanged briefly and needed to be restarted. In this case, none of the elution pools were collected from the chromatography columns during the time of the deviations in the BioSMB valve manifold. The operating system was paused and the open/close positions of all valves were manually checked. The impact on the overall process was mitigated by rapidly switching columns and not allowing any deviated elution material to mix with well-controlled material. However, for the part of the elution which was collected into the subsequent surge tank, the impact was that the concentration of that subsequent surge tank was reduced and its pH was deviated for a few cycles until the fluctuation dampened out. Due to the material loss on the column that was switched out, there was a reduction in the yield of the Protein A step for one cycle. Also, the binding onto the CEX columns was affected due to the pH deviations, and thus these CEX elution pools were not collected, lowering the process yield and affecting UFDF operation.

### 4.5. CEX Chromatography Deviations

Several deviations arose in the CEX chromatography operations. Firstly, there was an incorrect neutralization pH post-viral inactivation, which corresponded to the load of the CEX columns (Deviation 2 in Table 2). In this case, after the deviation was detected using the control charts, an alarm was sent to the operator and flow into the post-viral inactivation surge tank was paused for manual checks. The material was allowed to build up in the surge tank during the pause time (<10 min). The operator switched out the pump and checked the RS-232 connections, pump integrity, pump head operation, and tubing. The incorrect flow of pH neutralization solution was rectified and the operation was restarted.

The next deviation arose in the CEX elution gradient due to the emptying of one of the CEX buffer tanks (Deviation 3 in Table 2). Once the deviation was detected, the elution pools from the deviated CEX cycles were directed to waste, rather than to the next surge tank, by opening the waste valves on the BioSMB and pausing the inflow to the surge tank. The scheduling of the subsequent elution was put on hold until the operator rectified the error by refilling the buffer tank, after which the elution pools were again directed into the next surge tank. The overall impact of these deviations on the process was that the binding efficiency and yield of the CEX step were reduced, leading to a lower elution pool concentration and lower yield of CEX for a few cycles until the pH fluctuation dampened out. This later affected the performance of cycle 5 of the UFDF step, leading to Deviation 5. The surge tank strategy allowed the process to be paused to rectify the errors while allowing the material flow across the process to continue smoothly.

### 4.6. UFDF Deviations

The earlier deviations in the ProA and CEX unit operations led to a deviation arising in cycle 5 of the UFDF operations, which is the final operation in the continuous mAb processing train (Deviation 5 in Table 2). Due to these errors, the total material volume and concentration in the UFDF load tank were all lower than during the steady-state process. However, as this is the final step in the process, it is flexible to adjustments without impacting any subsequent unit’s operation. The operating time of the UFDF is determined by the weight of material in the load tank, as the end a UFDF cycle is triggered when the weight reduces to below a minimum set point. The lower concentration of starting material led to lower transmembrane and operating pressures for the UFDF cycle, which led to the deviation detected on the control charts. Overall, cycle 5 of the UFDF step was successfully executed based on surge tank triggers to compensate for the lower quantity of material.

From the deviation case studies above, it can be seen that the surge tank control framework used the process integration and scheduling in Module 1, combined with the results of the MVDA analyses in Module 2, to trigger a pop-up window with an alarm message to alert the operator of a deviation. At the same time, the loading to the concerned unit operation was immediately paused, and the outlet valves were directed to waste. The operator would then have time to manually examine the unit operation for errors and carry out repairs or replacements on the equipment, such as replacing tubing, recalibrating sensors, identifying leaks, resetting software, carrying out manual cleaning procedures, or replacing consumables such as columns, membranes, or filters in case of damage. Meanwhile, the previous unit operations would continue to operate until the surge tanks reached their set maximum weight points, after which they would pause in reverse order. Once the error was rectified, the operator would have the option to click on the alarm prompt to restart the train and input information about the deviation and the corrective actions undertaken, which would be stored with a time-stamped signature in the CPPS. The preceding and subsequent unit operations would restart automatically once their surge tanks reached acceptable weight ranges as the material once again began flowing across the continuous train.

## 5. Conclusions and Future Directions

The manufacturing of monoclonal antibodies is challenging due to the large number of complex unit operations that need to be undertaken. Biopharma products and processes are characterized by the presence of large number of CQAs and CPPs, respectively. All of these need to be monitored and controlled in order to produce a safe and efficacious mAb product. This complexity is a major impediment to the successful adoption of continuous processing in biopharma manufacturing, despite the significant advantages it offers in terms of process economics and manufacturing capacity. Major challenges in automation, process scheduling, process monitoring, and real-time process correction need to be overcome. Furthermore, CPV requires sophisticated systems for the storage, retrieval, and real-time analysis of data that can handle all the heterogenous sources of data from the process equipment, analytical instruments, in-line sensors, and operator actions, all of which need to be organized and converted from various file formats and locations (such as tabular files, images, spectra, distributed file systems, and run logs). Carrying out all these activities manually, including data logging, organization, monitoring, statistical analysis, process supervision, and real-time control, is not feasible over long continuous campaigns without introducing multiple errors. 

The case study presented in this work is the first end-to-end CPPS for the continuous manufacturing of mAbs. The proposed automated system has been successfully demonstrated to handle process integration, automation, data collection, storage, retrieval, analysis, monitoring, and control, automatically carrying out all these tasks to maintain the process at a steady state, bringing in operators only when corrective measures are required due to unexpected process breakdown. The CPPS includes the functionality of data acquisition followed by real-time data analysis, process monitoring, process supervision and real-time control actions. The system is in line with Industry 4.0 and smart manufacturing concepts and enables plug-and-play modules with different unit operations to be linked together via a system of surge tanks and integrated with a control system, virtual data analytics, and visualization tools, as well as the integration of real-time data acquisition with historical data to enable monitoring and control. By having surge tanks as intermediate vessels between the different unit operations, the process material can be redirected to waste as and when needed, without causing fluctuations in the feed flow to the next unit operation, which continues to smoothly draw material from the preceding tank. This also provided the flexibility to direct the outflow from any of the operations to waste without worrying about the operation of the other steps, as they would remain in a state of temporary pause after completing their ongoing cycle until their surge tanks again filled with the material equivalent to at least one cycle. The proposed CPPS has been deployed at the local level for a continuous train at lab scale, and there is future scope for implementing the solution at a plant-wide or company-wide level for the operation of multiple continuous processing campaigns across space and time with a unified control system enabling the tracking of operations, errors, the material produced, and forecasted demand.

Other obstacles currently being faced by the biopharmaceutical industry include inefficient routine manufacturing processes that operate in batch mode with a suboptimal utilization of consumables and space and result in frequent failed batches due to variability in a multidimensional set of process parameters, which affect critical quality attributes via mechanisms that are not completely understood. Converting these outdated manufacturing processes to a continuous mode has several challenges. Firstly, there is a shortage of trained people who not only have deep domain knowledge in the area of biopharmaceuticals, but are also skilled in process automation, hardware–software integration, programming, and advanced data analytics. Secondly, there is a lack of standardized interfaces for connected biopharmaceutical process equipment and few biopharma-specific tools for data analysis and modelling. Finally, there is a lack of guidelines and frameworks for the control of continuous processes from regulatory authorities. Thus, a CPPS could be a key enabler of continuous processing, providing an automation infrastructure and software product for data aggregation and management that enables the real-time analysis of an ongoing process using cutting-edge multivariate algorithms, and one which provides case-specific process controls based on surge tanks which enable deviated material volumes to be discarded and equipment parts to be replaced without stopping the continuous operation.

## Figures and Tables

**Table 1:** Hardware and software input–output protocols for the production platform.

	Unit Operation—Measurement	Hardware I/O	Software I/O
Bioreactor	Bioreactor pH, DO, CO_2_, temperature	x	RS-232
	Turbidity	x	Modbus 485
	Glucose	Analog	x
	Conductivity	x	Modbus 485
Clarification	Turbidity	x	Modbus 485
	Pressure	Digital	x
	Weight	x	RS-232
	Pump	Digital	X
	Solenoid valve	Digital	X
Chromatography	Turbidity	x	Modbus 485
	Weight	x	RS-232
	Pump	Digital	X
	pH	Digital	X
	HPLC	x	Java.net
	Pump	Digital	X
Viral Inactivation	Pressure	Digital	X
	Valve	Digital	X
	pH	Digital	X
Depth Filtration	Pressure	Digital	X
	Weight	Digital	X
	Solenoid valve	Digital	X
Formulation	Pressure	Digital	X
	Valves	Digital	X
	Weight	x	RS-232

**Table 2:** Deviations detected during continuous operation.

S. No.	Description of Deviation	Cycle(s) Affected	Data Source Allowing Detection of the Deviation
1	Pressure build-up and leakage in one of the Protein A columns due to tubing compression	Chromatography cycles 43, 44, 45	Chromatography PCA charts (Figure 6a,b) taking inputs from Protein A UV chromatogram (Figure 5c, Protein A elution pool)
2	Deviation in the pH neutralization step after viral inactivation due to error in the pump supplying pH neutralization solution	Chromatography cycles 43, 44, 45	Chromatography PCA charts (Figure 6a,b) taking inputs from BioSMB pH sensor data (Figure 5b, post-viral inactivation)
3	Deviation in CEX elution gradient due to emptying of one of the buffer tanks	Chromatography cycles 45, 46, 47	Chromatography PCA charts (Figure 6a,b) taking inputs from BioSMB conductivity sensor data (Figure 5a, conductivity A and B)
4	Deviation in the valve open/close positions on the BioSMB valve manifold due to controller error	Chromatography cycles 50, 51	Chromatography PCA charts (Figure 6a,b) taking inputs from BioSMB pH sensor data (Figure 5b, pre-viral inactivation)
5	Deviation in process volume and concentration in the UFDF feed tank due to deviations in the preceding unit operations	UFDF cycle 5	UFDF PCA charts (Figure 6d,e) taking inputs from UFDF pressure sensor data (Figure 5d feed, retentate, and permeate pressures)

## Data Availability

Data will be made available on request.

## Supplementary Materials

The following supporting information can be downloaded at: https://www.mdpi.com/article/10.3390/bioengineering11060610/s1, S1: Pre-processing of spectral data; S2: Principal Component Analysis.
