# Supplementary material for: A Cyber–Physical Production System for the Integrated Operation and Monitoring of a Continuous Manufacturing Train for the Production of Monoclonal Antibodies
Source: Bioengineering (Basel). 2024 Jun 13;11(6):610. doi: 10.3390/bioengineering11060610 (PMC11200404; doi:10.3390/bioengineering11060610)
Supplement: Supplementary file 1 [file bioengineering-11-00610-s001.zip › bioengineering-2973421-supplementary.pdf]

## Supplementary Information

## S1: Pre-processing of spectral data

The Savitzky-Golay algorithm is used as the smoothing algorithm used to remove noise from data, particularly in signal processing and time-series analysis. It works by fitting a polynomial of a specified degree to a window of adjacent data points and then using the coefficients of this polynomial to estimate the smoothed value at the central point of the window.

Input- real time data from different unit operations

Output - smoothened data for analysis

Pseudocode -

1. Window Size Selection: Choose a window size n, typically an odd integer, which determines the number of adjacent data points used for smoothing.

2. Polynomial Fitting: Within each window, fit a polynomial of a specified degree to the data points. The polynomial is usually a low-degree polynomial to avoid overfitting.

3. Solving Coefficients: Solve for the coefficients of the polynomial using least squares regression. This involves minimizing the squared differences between the actual data points and the values predicted by the polynomial.

4. Smoothing: Use the coefficients obtained from the polynomial fitting to estimate the smoothed value at the central point of the window.

5. Moving Window: Slide the window across the entire dataset, repeating the polynomial fitting and smoothing process at each position.

The Savitzky-Golay algorithm effectively smoothes the data while preserving important features such as peaks and valleys. It is often used in applications where noise reduction is critical, such as in spectroscopy, chromatography, and biomedical signal processing.

## S2: Principal Component Analysis

Principal component analysis is used as the MVDA algorithm. Principal Component Analysis (PCA) is a dimensionality reduction technique commonly used in data analysis and machine learning. It transforms high-dimensional data into a new coordinate system where the greatest variance lies on the first coordinate (principal component), the second greatest variance on the second coordinate, and so forth. This process allows for the reduction of data complexity while preserving as much of the original variance as possible. PCA is widely employed in various fields such as image processing, feature extraction, and exploratory data analysis.

Input: A dataset X consisting of m observations (samples) and n features (variables). Along with the desired number of principal components k.

Output: The principal components, which are the new orthogonal basis vectors, transformed data, where each observation is represented in terms of the principal components, and the eigenvalues corresponding to each principal component, which represent the amount of variance explained by each component.

Pseudocode:

1. Center the Data: Subtract the mean of each feature from the corresponding feature values in the dataset X, resulting in a centered dataset

2. Compute Covariance Matrix: Compute the covariance matrix of the centered data.

3. Compute Eigenvalues and Eigenvectors: Compute the eigenvalues and eigenvectors of the covariance matrix

4. Select Principal Components: Select the top k eigenvectors corresponding to the k largest eigenvalues to retain k principal components.

5. Transform Data: Project the centered data onto the selected principal components to obtain the transformed data

6. Return Output: Return the principal components and the transformed data
